# Supplementary material for: PSAT1 Promotes Metastasis via p-AKT/SP1/ITGA2 Axis in Estrogen Receptor-Negative Breast Cancer Cell
Source: Biomolecules. 2024 Aug 12;14(8):990. doi: 10.3390/biom14080990 (PMC11352415; doi:10.3390/biom14080990)
Supplement: Supplementary file 1 [file biomolecules-14-00990-s001.zip › Tables S1-S4.pdf]

Table S1: Reagents and resource used in this study.

KEY RESOURCES TABLE		
REAGENT or RESOURCE	SOURCE	IDENTIFIER
Antibodies		
PSAT1	Genetex	GTX110576
ITGA2	Abcam	ab133557
ITGA2	Santa	#C1022
p-AKT	Cell Signaling Technology	Antibody #9271
SP1	Proteintach	21962-1-AP
SP1	Genetex	GTX110593
Actin	Proteintech	66009-1-Ig
Tubulin	Abclonal	AC021
Lentivirus, siRNA and plasmid		
LV-PSAT1-RNAi(Human)	GeneChem	
LV-Psat1(Human)	GeneChem	
LV-PSAT1-RNAi(Mouse)	GeneChem	
LV-Psat1(Mouse)	GeneChem	
siPSAT1	RiboBio	tB0011000B/tB0011000C
siITGA2	RiboBio	tB0001166C
pLVX-puro-PSAT1	Clontech	
Inhibitor		
LY294002	Medchemexpress	HY-10108
Mithramycin A	Medchemexpress	HY-A0122
Kit		
CHIP Assay Kit	Beyotime Biotechnology	P2078
Plasmid Kit	Tiangen	DP103
Nuclear and Cytoplasmic Protein Extraction Kit	Wanleibio	WLA020

Table S2: The sequences for lentivirus and all siRNAs.

lentivirus	
PSAT1-RNAi#1	TTCCAAGTTTGGTGTGATT
PSAT1-RNAi#2	ACTCAGTGTTGTTAGAGAT
siRNAs	
si-PSAT1#1	GGTGCATGGTGTGGAGTTT
si-PSAT1#2	GAAACAGCTCCTTGTACAA
si-ITGA2#1	GTAGCAACATCCCAGACAT

Table S3: The sequences for all primers.

PSAT1-F(H)	5'-GTCCAGTGGAGCCCCAAAA-3'
PSAT1-R(H)	5'-TGCCTCCCACAGACCTATGC-3'
ITGA2-F(H)	5'-CTCCTAAACTCAGTCAT-3'
ITGA2-R(H)	5'-CACAAGTTATGATTATCC-3'
LAMA5-F(H)	5'- GGACCTCTACTGCAAGCTGGT-3'
LAMA5-R(H)	5'- ATAGGCCACATGGAACACCTG-3'
CLDN3-F(H)	5'- CCACGCGAGAAGAAGTACACG-3'
CLDN3-R(H)	5'- AGACGTAGTCCTTGCGGTCGTA-3'
CLDN7-F(H)	5'- TTTTCATCGTGGCAGGTCTTG-3'
CLDN7-R(H)	5'- CCCTGCCCAGCCAATAAAGA-3'
UUP1-F(H)	5'- AACAGAGCAGGCAGTGGATA-3'
UUP1-R(H)	5'- ATACGCCTGCTTGTCCTCTT-3'
β-actin-F(H)	5'-CATGTACGTTGCTATCCAGGC-3'
β-actin-R(H)	5'-CTCCTTAATGTCACGCACGAT-3'
PSAT1-F(M)	5'-AAGCCACCAAGCAAGTGGTTA-3'
PSAT1-R(M)	5'-GATGCCGAGTCCTCTGTAGTC-3'
ITGA2-F(M)	5'-TGTCTGGCGTATAATGTTGGC-3'
ITGA2-R(M)	5'-CTTGTGGGTTCGTAAGCTGCT-3'
β-actin-F(M)	5'-GGCTGTATTCCCCTCCATCG-3'
β-actin-R(M)	5'-CCAGTTGGTAACAATGCCATGT-3'

Table S4: The binding sites of SP1 to ITGA2 predicted by JASPER.

Matrix ID	Name	Score	Relative score	Sequence ID	Start	End	Strand	Predicted sequence
MA0079.1	MA0079.1.SP1	8.273666	0.875462	NC_000005.10:52989352-52991352	312	321	-	GTGGCTGGGA
MA0079.2	MA0079.2.SP1	9.760581	0.877109	NC_000005.10:52989352-52991352	1827	1836	+	TCCCTCCTCC
MA0079.2	MA0079.2.SP1	9.087278	0.860152	NC_000005.10:52989352-52991352	806	815	-	CCCCTCTGCC
MA0079.2	MA0079.2.SP1	9.060908	0.859488	NC_000005.10:52989352-52991352	978	987	+	GCCCGCCCAC
MA0079.3	MA0079.3.SP1	12.02735	0.932458	NC_000005.10:52989352-52991352	1826	1836	+	TTCCCTCCTCC
MA0079.3	MA0079.3.SP1	11.60073	0.927091	NC_000005.10:52989352-52991352	977	987	+	GGCCCGCCCAC
MA0079.3	MA0079.3.SP1	9.758064	0.903908	NC_000005.10:52989352-52991352	1026	1036	-	TCTCCACCCCA
MA0079.3	MA0079.3.SP1	9.481408	0.900428	NC_000005.10:52989352-52991352	981	991	+	CGCCCACCTCC
MA0079.3	MA0079.3.SP1	9.366858	0.898986	NC_000005.10:52989352-52991352	1829	1839	+	CCTCCTCCTAC
MA0079.3	MA0079.3.SP1	9.189689	0.896757	NC_000005.10:52989352-52991352	1009	1019	-	TTTCCTCCTCT
MA0079.3	MA0079.3.SP1	8.446419	0.887406	NC_000005.10:52989352-52991352	253	263	-	GTTCCGCCCAA
MA0079.4	MA0079.4.SP1	7.201846	0.86053	NC_000005.10:52989352-52991352	974	988	+	TGGGGCCCGCCCACC
MA0079.5	MA0079.5.SP1	8.464037	0.865965	NC_000005.10:52989352-52991352	1027	1035	+	GGGGTGGAG
MA0079.5	MA0079.5.SP1	8.398116	0.864819	NC_000005.10:52989352-52991352	254	262	+	TGGGCGGAA
