# Supplementary figures and images for: PSAT1 Promotes Metastasis via p-AKT/SP1/ITGA2 Axis in Estrogen Receptor-Negative Breast Cancer Cell
Source: Biomolecules. 2024 Aug 12;14(8):990. doi: 10.3390/biom14080990 (PMC11352415; doi:10.3390/biom14080990)

Figure S1

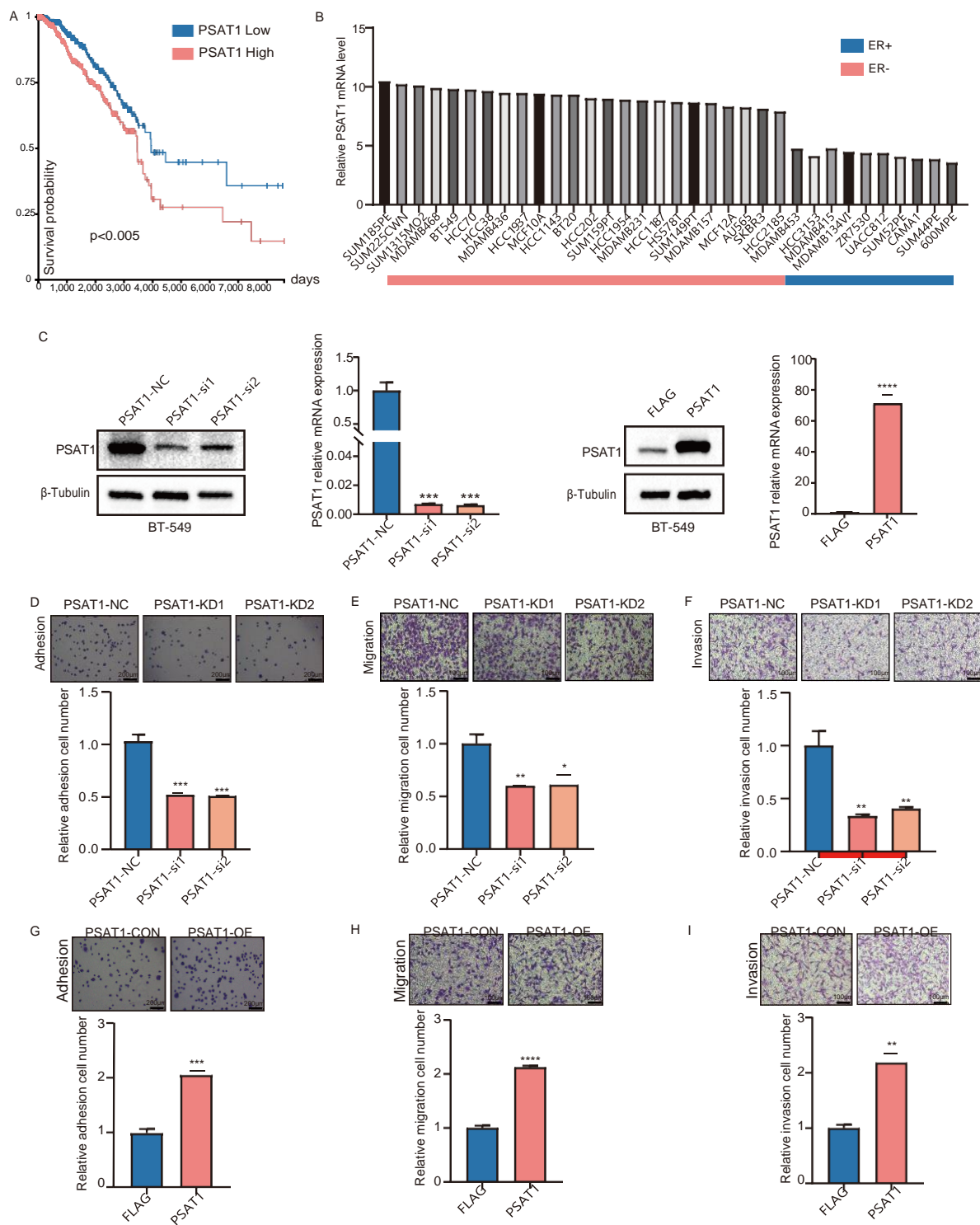

Figure S2

A

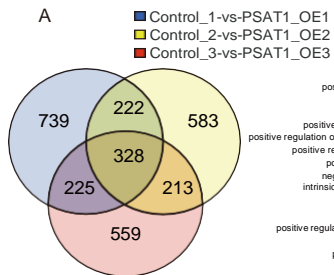

C

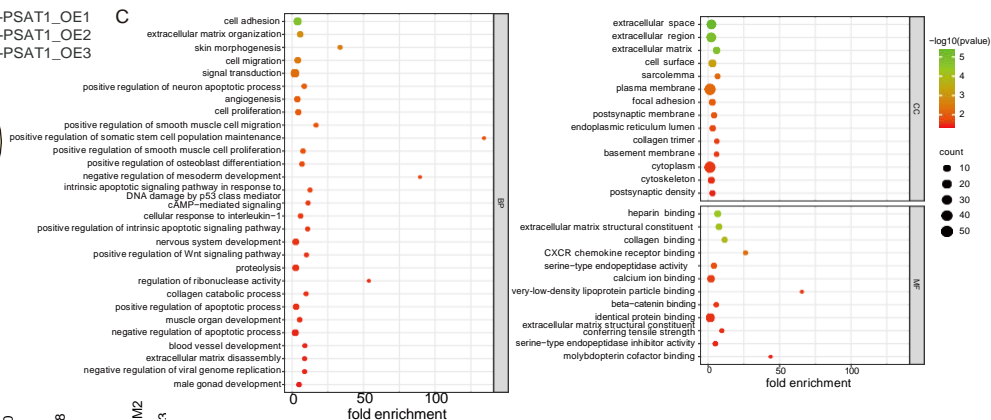

B

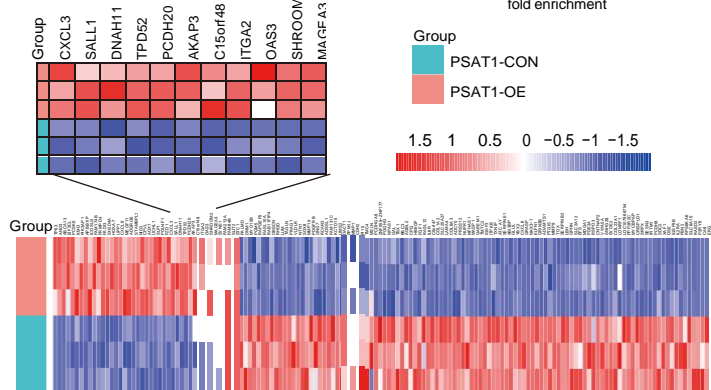

D

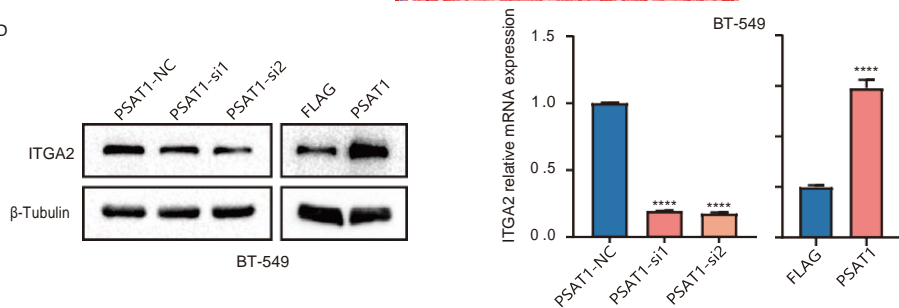

E

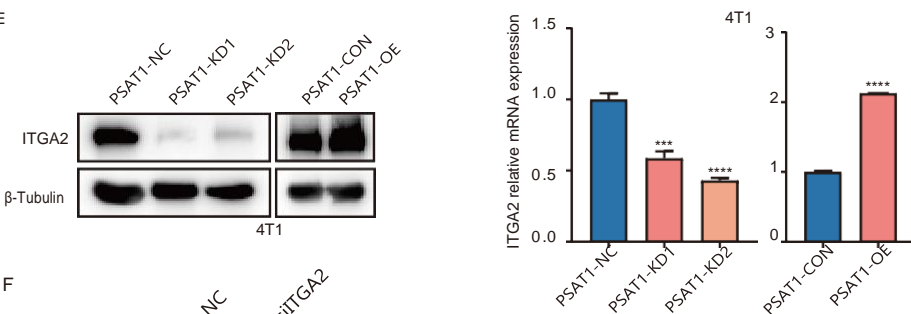

F

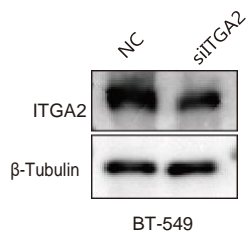

Supplement: Supplementary file 1 [file biomolecules-14-00990-s001.zip › Figures S1 and S2.pdf]
